# 1,4-Diphenyl­butane-1,4-dione

**DOI:** 10.1107/S1600536808038798

**Published:** 2008-11-22

**Authors:** Zhigang Wang

**Affiliations:** a School of Chemical and Materials Engineering, Huangshi Institute of Technology, Huangshi 435003, People’s Republic of China

## Abstract

The asymmetric unit of the title compound, C_16_H_14_O_2_, contains one half-mol­ecule, located on a twofold rotation axis. In the mol­ecule, the two benzene rings form a dihedral angle of 72.28 (2)°.

## Related literature

For useful applications of 1,4-dicarbonyl compounds, see: Chiu & Sammes (1990 ▶); Greatrex *et al.* (2003 ▶); Nagarajan & Shechter (1984 ▶). For details of the synthesis, see Nevar *et al.* (2000 ▶).
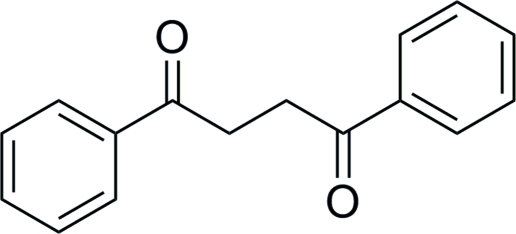


## Experimental

### 

#### Crystal data

C_16_H_14_O_2_
*M* *_r_* = 238.27
Orthorhombic, 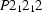

*a* = 8.3781 (13) Å
*b* = 14.161 (2) Å
*c* = 5.3186 (8) Å
*V* = 631.00 (17) Å^3^
*Z* = 2
Mo *K*α radiation
μ = 0.08 mm^−1^
*T* = 298 (2) K
0.20 × 0.10 × 0.10 mm

#### Data collection

Bruker SMART CCD area-detector diffractometer
Absorption correction: multi-scan (*SADABS*; Sheldrick, 1997 ▶) *T*_min_ = 0.984, *T*_max_ = 0.992
4063 measured reflections
762 independent reflections
640 reflections with *I* > 2σ(*I*)
*R*_int_ = 0.163

#### Refinement

*R*[*F* ^2^ > 2σ(*F* ^2^)] = 0.054
*wR*(*F* ^2^) = 0.129
*S* = 1.05
762 reflections
83 parameters
H-atom parameters constrained
Δρ_max_ = 0.20 e Å^−3^
Δρ_min_ = −0.22 e Å^−3^

### 

Data collection: *SMART* (Bruker, 1997 ▶); cell refinement: *SAINT* (Bruker, 1999 ▶); data reduction: *SAINT*; program(s) used to solve structure: *SHELXS97* (Sheldrick, 2008 ▶); program(s) used to refine structure: *SHELXL97* (Sheldrick, 2008 ▶); molecular graphics: *SHELXTL* (Sheldrick, 2008 ▶); software used to prepare material for publication: *SHELXTL*.

## Supplementary Material

Crystal structure: contains datablocks I, global. DOI: 10.1107/S1600536808038798/cv2479sup1.cif

Structure factors: contains datablocks I. DOI: 10.1107/S1600536808038798/cv2479Isup2.hkl

Additional supplementary materials:  crystallographic information; 3D view; checkCIF report

## Figures and Tables

**Fig. 1.**
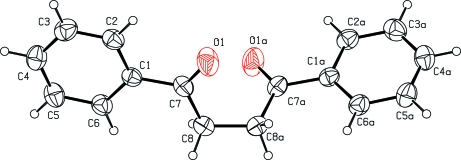
View of the title molecule showing the atom-labelling scheme. The displacement ellipsoids are drawn at the 30% probability level [symmetry code: (a) -x, -y + 1, z].

## supplementary crystallographic information

### Comment

1,4-Dicarbonyl compounds constitute key intermediates in various natural product syntheses, and they are important synthetic precursors of cyclopentenones, cyclopenta-1,3-diones, butenolides, and derivatives of furan and pyrrole (Chiu & Sammes, 1990; Greatrex *et al.*, 2003; Nagarajan & Shechter, 1984). Herewith we present the title compound (I) (Fig. 1). The asymmetric unit of (I) contains a half of the molecule located on a twofold rotational axis. Two benzene rings form a dihedral angle of 72.28 (2)°.

### Experimental

The title compound was synthesized as previously described by Nevar *et al.* (2000). Colourless crystals suitable for X-ray data collection were obtained by slow evaporation of a 1:3 ratio EtOAc:cyclohexane solution at room temperture.

### Refinement

All H atoms were positioned geometrically (C—H = 0.93–0.97 Å) and refined as riding, allowing for free rotation of the methyl groups. The constraint *U*_iso_(H) = 1.2 *U*_eq_(C) or 1.5 *U*_eq_(C) (methyl C) was applied. In the absence of anomalous scatterers, no attempt was made to establish the absolute configuration of the title compound, and 488 Friedel pairs were merged before the final refinement.

### Crystal data

**Table:**

C_16_H_14_O_2_	*F*_000_ = 252
*M**_r_* = 238.27	*D*_x_ = 1.254 Mg m^−^^3^
Orthorhombic, *P*2_1_2_1_2	Mo *K*α radiation λ = 0.71073 Å
Hall symbol: P 2 2ab	Cell parameters from 1423 reflections
*a* = 8.3781 (13) Å	θ = 2.8–22.3º
*b* = 14.161 (2) Å	µ = 0.08 mm^−^^1^
*c* = 5.3186 (8) Å	*T* = 298 (2) K
*V* = 631.00 (17) Å^3^	Block, colourless
*Z* = 2	0.20 × 0.10 × 0.10 mm

### Data collection

**Table:**

Bruker SMART CCD area-detector diffractometer	762 independent reflections
Radiation source: fine-focus sealed tube	640 reflections with *I* > 2σ(*I*)
Monochromator: graphite	*R*_int_ = 0.163
*T* = 298(2) K	θ_max_ = 26.0º
φ and ω scans	θ_min_ = 2.8º
Absorption correction: multi-scan(SADABS; Sheldrick, 1997)	*h* = −10→9
*T*_min_ = 0.984, *T*_max_ = 0.992	*k* = −16→17
4063 measured reflections	*l* = −6→6

### Refinement

**Table:**

Refinement on *F*^2^	Secondary atom site location: difference Fourier map
Least-squares matrix: full	Hydrogen site location: inferred from neighbouring sites
*R*[*F*^2^ > 2σ(*F*^2^)] = 0.054	H-atom parameters constrained
*wR*(*F*^2^) = 0.129	*w* = 1/[σ^2^(*F*_o_^2^) + (0.0734*P*)^2^] where *P* = (*F*_o_^2^ + 2*F*_c_^2^)/3
*S* = 1.05	(Δ/σ)_max_ = 0.012
762 reflections	Δρ_max_ = 0.20 e Å^−^^3^
83 parameters	Δρ_min_ = −0.22 e Å^−^^3^
Primary atom site location: structure-invariant direct methods	Extinction correction: none

### Special details

**Table:**

Geometry. All e.s.d.'s (except the e.s.d. in the dihedral angle between two l.s. planes) are estimated using the full covariance matrix. The cell e.s.d.'s are taken into account individually in the estimation of e.s.d.'s in distances, angles and torsion angles; correlations between e.s.d.'s in cell parameters are only used when they are defined by crystal symmetry. An approximate (isotropic) treatment of cell e.s.d.'s is used for estimating e.s.d.'s involving l.s. planes.
Refinement. Refinement of *F*^2^ against ALL reflections. The weighted *R*-factor *wR* and goodness of fit *S* are based on *F*^2^, conventional *R*-factors *R* are based on *F*, with *F* set to zero for negative *F*^2^. The threshold expression of *F*^2^ > σ(*F*^2^) is used only for calculating *R*-factors(gt) *etc*. and is not relevant to the choice of reflections for refinement. *R*-factors based on *F*^2^ are statistically about twice as large as those based on *F*, and *R*- factors based on ALL data will be even larger.

### Fractional atomic coordinates and isotropic or equivalent isotropic displacement parameters (Å^2^)

**Table:**

	*x*	*y*	*z*	*U*_iso_*/*U*_eq_	
C1	0.1095 (3)	0.30567 (15)	0.2151 (4)	0.0508 (6)	
C7	0.1070 (3)	0.40988 (16)	0.1653 (5)	0.0574 (7)	
C4	0.1208 (3)	0.11385 (19)	0.3179 (6)	0.0699 (7)	
H4	0.1241	0.0496	0.3532	0.084*	
C8	0.0072 (4)	0.44647 (16)	−0.0468 (5)	0.0670 (7)	
H8A	−0.0986	0.4191	−0.0354	0.080*	
H8B	0.0540	0.4263	−0.2047	0.080*	
C3	0.2031 (3)	0.17678 (17)	0.4670 (5)	0.0704 (8)	
H3	0.2625	0.1548	0.6026	0.085*	
C6	0.0283 (3)	0.24197 (17)	0.0654 (5)	0.0608 (7)	
H6	−0.0307	0.2634	−0.0713	0.073*	
C2	0.1980 (3)	0.27214 (16)	0.4163 (5)	0.0608 (7)	
H2	0.2541	0.3142	0.5176	0.073*	
O1	0.1843 (3)	0.46221 (13)	0.2966 (5)	0.0984 (8)	
C5	0.0345 (3)	0.14619 (18)	0.1188 (6)	0.0712 (8)	
H5	−0.0206	0.1037	0.0177	0.085*	

### Atomic displacement parameters (Å^2^)

**Table:**

	*U*^11^	*U*^22^	*U*^33^	*U*^12^	*U*^13^	*U*^23^
C1	0.0464 (11)	0.0571 (13)	0.0490 (11)	0.0054 (10)	0.0025 (10)	−0.0053 (10)
C7	0.0558 (13)	0.0561 (13)	0.0603 (14)	0.0011 (11)	−0.0034 (12)	−0.0105 (11)
C4	0.0636 (15)	0.0595 (13)	0.0867 (18)	0.0061 (13)	0.0053 (15)	0.0043 (15)
C8	0.0839 (17)	0.0614 (14)	0.0555 (13)	0.0048 (13)	−0.0040 (14)	−0.0074 (11)
C3	0.0658 (16)	0.0769 (17)	0.0686 (16)	0.0155 (14)	−0.0023 (15)	0.0091 (15)
C6	0.0613 (14)	0.0604 (13)	0.0607 (13)	−0.0011 (11)	−0.0092 (13)	−0.0065 (12)
C2	0.0560 (14)	0.0676 (14)	0.0589 (14)	0.0046 (12)	−0.0060 (12)	−0.0094 (12)
O1	0.1149 (17)	0.0632 (11)	0.1172 (17)	−0.0067 (11)	−0.0544 (16)	−0.0120 (12)
C5	0.0696 (17)	0.0597 (14)	0.0843 (17)	−0.0059 (13)	−0.0060 (16)	−0.0117 (14)

### Geometric parameters (Å, °)

**Table:**

C1—C6	1.382 (3)	C8—H8A	0.9700
C1—C2	1.386 (3)	C8—H8B	0.9700
C1—C7	1.499 (3)	C3—C2	1.378 (3)
C7—O1	1.207 (3)	C3—H3	0.9300
C7—C8	1.496 (3)	C6—C5	1.387 (4)
C4—C5	1.361 (4)	C6—H6	0.9300
C4—C3	1.378 (3)	C2—H2	0.9300
C4—H4	0.9300	C5—H5	0.9300
C8—C8^i^	1.521 (4)		
			
C6—C1—C2	119.0 (2)	H8A—C8—H8B	107.8
C6—C1—C7	122.3 (2)	C4—C3—C2	120.4 (2)
C2—C1—C7	118.74 (19)	C4—C3—H3	119.8
O1—C7—C8	121.5 (2)	C2—C3—H3	119.8
O1—C7—C1	119.7 (2)	C1—C6—C5	120.1 (2)
C8—C7—C1	118.8 (2)	C1—C6—H6	119.9
C5—C4—C3	119.7 (2)	C5—C6—H6	119.9
C5—C4—H4	120.1	C3—C2—C1	120.2 (2)
C3—C4—H4	120.1	C3—C2—H2	119.9
C7—C8—C8^i^	112.9 (2)	C1—C2—H2	119.9
C7—C8—H8A	109.0	C4—C5—C6	120.6 (2)
C8^i^—C8—H8A	109.0	C4—C5—H5	119.7
C7—C8—H8B	109.0	C6—C5—H5	119.7
C8^i^—C8—H8B	109.0		
			
C6—C1—C7—O1	−177.5 (3)	C2—C1—C6—C5	0.7 (3)
C2—C1—C7—O1	1.7 (4)	C7—C1—C6—C5	179.8 (3)
C6—C1—C7—C8	2.8 (3)	C4—C3—C2—C1	0.2 (4)
C2—C1—C7—C8	−178.0 (2)	C6—C1—C2—C3	−0.6 (4)
O1—C7—C8—C8^i^	−9.8 (4)	C7—C1—C2—C3	−179.9 (2)
C1—C7—C8—C8^i^	169.9 (3)	C3—C4—C5—C6	−0.3 (4)
C5—C4—C3—C2	0.3 (4)	C1—C6—C5—C4	−0.2 (4)

Symmetry codes: (i) −*x*, −*y*+1, *z*.
